# The Gut–Vascular Axis as a Therapeutic Hub: Rationale and Evidence for Combined Gut-Directed and Endothelium-Directed Interventions

**DOI:** 10.3390/metabo16100780

**Published:** 2026-10-14

**Authors:** Tianyi Gu, Ruochen Wang, Shengyu Hua

**Affiliations:** College of Traditional Chinese Medicine, Tianjin University of Traditional Chinese Medicine, Tianjin 301617, China

**Keywords:** gut–vascular axis, intestinal barrier, vascular endothelial function, microbiota, combined intervention, therapeutic targets

## Abstract

Background: The gut–vascular axis linking gut microbiota, intestinal barrier, and vascular endothelium is a key pathophysiological interface in chronic metabolic diseases. Dysbiosis and “leaky gut” enable bacterial products and gut-derived metabolites to enter the circulation, driving systemic inflammation and endothelial dysfunction. Single-target interventions show limited efficacy; combined gut-directed and endothelium-directed strategies remain underexplored. Methods: PubMed was searched from database inception to 31 August 2026 using a predefined search strategy combining terms for the gut–vascular axis, intestinal barrier, microbiota/metabolites, endothelial dysfunction, and combined interventions. Preclinical and clinical studies evaluating gut barrier integrity, microbiota modulation, or endothelial protection, alone or in combination, were included. No formal risk-of-bias scoring was performed; study design, sample size, endpoint type, follow-up, and limitations were appraised. Results: (1) The axis comprises three core components: gut barrier, endothelium, and microbial metabolites. (2) Pathological activation involves leaky-gut-driven endotoxemia, dysbiosis-induced metabolic inflammation, and immune crosstalk sustaining vascular inflammation. (3) Animal models demonstrate that combined interventions can reduce atherosclerotic plaque, improve endothelium-dependent relaxation, and lower systemic inflammation to a greater extent than some monotherapies; however, most studies have not directly compared combination therapy with both corresponding monotherapies or performed formal interaction testing, so true synergy remains unproven. (4) Limited clinical studies in metabolic syndrome and heart failure suggest improvements in surrogate endpoints, but large-scale randomised trials with hard cardiovascular endpoints are lacking. Conclusions: The gut–vascular axis is a promising therapeutic framework for cardiometabolic diseases, with a strong biological rationale. Combined gut- and endothelium-directed interventions offer a rational multi-target approach, but direct evidence that they provide superior clinical outcomes remains limited. Future research must prioritise hard-endpoint trials, standardised interventions and endpoints, long-term safety, reproducibility, multi-omics-based patient stratification, and next-generation microbiome therapies.

## 1. Introduction

The gut–vascular axis (GVA) is defined here as the bidirectional, multi-level communication network that functionally connects the intestinal microenvironment with the systemic vascular system. Its main components are: (i) the gut microbiota and its metabolites, including protective short-chain fatty acids (SCFAs) and harmful trimethylamine N-oxide (TMAO), secondary bile acids, and tryptophan derivatives; (ii) the intestinal barrier, comprising the epithelial barrier and the deeper gut–vascular barrier (GVB); (iii) the vascular endothelium, including its glycocalyx and intercellular junctions; and (iv) gut-associated immune cells and circulating inflammatory mediators that relay signals between the gut and the vessel wall.

The gut is not only the central organ for digestion and absorption, but also a dynamic immunometabolic interface comprising a complex microbial ecosystem, a tightly regulated intestinal epithelial barrier, and a rich network of gut-associated lymphoid tissue, which profoundly influences systemic health [1]. The homeostasis of this interface is crucial for maintaining the function of distant organs, including the central nervous system: when intestinal homeostasis is disrupted—that is, when dysbiosis occurs—it is often accompanied by impaired intestinal barrier integrity, resulting in a state known as ‘leaky gut’ [2]. At this point, bacterial metabolites (such as lipopolysaccharides), pathogen-associated molecular patterns, and inflammatory mediators from the gut can translocate into the circulatory system [1]. This translocation occurs not only at the intestinal epithelial barrier but may also involve the deeper intestinal vascular barrier, which is composed of endothelial cells, pericytes and glial cells, and is specifically responsible for regulating the entry of macromolecules into the bloodstream [3]. Once the function of the intestinal vascular barrier is compromised, microorganisms or their metabolites can spread systemically, forming a key link between pathological changes in the intestine and those in distant organs [4].

These gut-derived substances entering the circulation directly or indirectly attack the vascular endothelium, triggering endothelial dysfunction, oxidative stress and chronic low-grade inflammation, which constitute the common pathological basis for a range of diseases including atherosclerosis, hypertension, type 2 diabetes and chronic kidney disease [5,6]. This body of evidence collectively points to a core concept: namely, that there exists a profound and bidirectional interactive network between the intestinal microenvironment and the vascular system, which can be summarised as the ‘gut–vascular axis’.

What this review adds to the existing literature is as follows. Although previous studies have separately addressed gut microbiota, intestinal barrier function, microbial metabolites, or endothelial dysfunction, this review integrates these findings into a single therapeutic framework centred on the GVA. It analyzes the core components and interactive mechanisms of the axis, evaluates the existing preclinical and clinical evidence for combined gut-directed and endothelium-directed interventions, and outlines the challenges and opportunities facing their clinical translation. In particular, it emphasises that the GVA is not merely a pathophysiological link but a therapeutic hub, and that combined interventions may act through source control at the gut level and downstream protection at the endothelial level.

The rationale for focusing on combined gut- and endothelial-targeted interventions is that single-target interventions show limited efficacy in this multi-node, bidirectional, and self-amplifying axis. Dysbiosis and leaky gut enable continuous translocation of LPS, TMAO, and inflammatory mediators into the circulation, driving systemic inflammation and endothelial dysfunction [7,8,9,10]; in turn, endothelial dysfunction and vascular inflammation can amplify the vicious cycle linking gut dysfunction to vascular lesions [11,12]. Treating only the gut may leave residual endothelial injury and impaired repair capacity, whereas treating only the endothelium may leave the gut-derived source of damage unresolved. Therefore, simultaneously restoring gut homeostasis and improving endothelial function—by blocking the source of gut-derived damaging factors and enhancing the vasculature’s own defensive and reparative capabilities—offers a rational multi-target approach [8,10]. This combined strategy aims to break the vicious cycle of “leaky gut–systemic inflammation–vascular lesions” through multi-level inhibition of inflammatory pathways such as TLR4/NF-κB, and may produce effects beyond monotherapy.

Based on this theoretical framework, bidirectional modulation of the gut–vascular axis—that is, simultaneously restoring gut homeostasis and improving endothelial function—is regarded as a highly promising new therapeutic paradigm [13]. This combined intervention strategy overcomes the limitations of traditional single-target approaches, aiming to block, at the source, the continuous assault of gut-derived damaging factors on the vascular system, whilst enhancing the vasculature’s own defensive and reparative capabilities. This review will provide an in-depth analysis of the core components and interactive mechanisms of this axis, critically summarise the existing evidence for combined intervention strategies, and outline the challenges and opportunities facing their clinical translation.

## 2. The Core Components and Physiological Basis of the Gut–Vascular Axis

### 2.1. The Intestinal Barrier: Structural and Functional Integrity

The intestinal barrier is a complex structure comprising multiple layers of defence mechanisms. Its core function is to ensure selective permeability, allowing the absorption of nutrients whilst effectively preventing harmful substances—such as bacteria and endotoxins—from translocating from the intestinal lumen into the systemic circulation [14]. This barrier system is primarily constituted by the synergistic action of mechanical, chemical, immune and biological barriers.

At the heart of the mechanical barrier lie the tight junctions between intestinal epithelial cells, which consist of transmembrane proteins such as occludin and the claudin family, as well as cytoplasmic scaffolding proteins such as zonula occludens-1 (ZO-1). Together, these form a physical seal that regulates the permeability of the intercellular spaces [15]. The chemical barrier comprises the mucus layer secreted by goblet cells, as well as antimicrobial peptides and secretory immunoglobulin A (sIgA) produced by Paneth cells and epithelial cells; together, these constitute the first line of chemical defence against pathogen colonisation and invasion [16]. The immune barrier involves a rich network of immune cells within the gut, such as dendritic cells, macrophages and lymphocytes, which are capable of recognising and eliminating pathogens that breach the physical and chemical barriers [17]. The biological barrier, meanwhile, is constituted by the vast symbiotic microbiota within the gut, which inhibits the growth of pathogenic bacteria by competing for nutrients, occupying ecological niches and producing metabolites (such as short-chain fatty acids), whilst actively participating in the regulation of barrier function [18].

The integrity of the intestinal barrier is not static, but rather exists in a state of dynamic equilibrium and renewal involving multiple cell types. Intestinal stem cells, located at the base of the intestinal crypts, continuously differentiate into various functional intestinal epithelial cells, including absorptive enterocytes, goblet cells, Paneth cells and enteroendocrine cells, which collectively maintain the integrity and function of the epithelium [19]; Paneth cells play a key role in innate immune defence at the base of the crypts by secreting antimicrobial peptides [16]; mucin proteins (such as MUC2) secreted by goblet cells form a gel-like layer covering the epithelial surface, effectively isolating the commensal microbiota from potential pathogens [20].

These cell functions are influenced by dietary components and microbial metabolites, although the strength of evidence differs across mechanisms. For example, butyrate and other SCFAs have been reported in in vitro and animal studies to enhance the expression and function of tight junction proteins and to strengthen the intestinal barrier [21]. Enteroendocrine cells have been proposed to contribute to barrier homeostasis through the secretion of hormones such as peptide YY (PYY) and somatostatin. Preclinical evidence indicates that these hormones may modulate intestinal barrier permeability, particularly under inflammatory stimulation [22]. However, the available data are limited, and their precise role in human gut–vascular axis regulation remains to be established.

Impaired barrier integrity is the initial key event that triggers pathological signalling in the gut–vascular axis. When mechanical, chemical or immune barrier functions are compromised, intestinal permeability increases abnormally, resulting in a state known as ‘leaky gut’ [23]. This allows substances that should normally be confined to the intestinal lumen—such as bacterial lipopolysaccharides (LPS), bacterial fragments, food antigens and microbial metabolites—to pass through the damaged intestinal epithelium into the portal venous circulation and systemic circulation [24]. This abnormal translocation of intestinal contents triggers systemic low-grade inflammation and immune activation. For example, LPS entering the circulation can activate Toll-like receptor 4 (TLR4) on immune cells, leading to the release of pro-inflammatory cytokines (such as tumour necrosis factor-α, TNF-α); these inflammatory factors, in turn, further disrupt the structure and function of tight junction proteins, creating a self-amplifying vicious cycle [25]. Consequently, intestinal barrier dysfunction is not only a hallmark of localised intestinal disease but also a precipitating factor for many systemic inflammatory diseases and vascular pathologies, providing a direct theoretical basis for the pathological link within the gut–vascular axis [16].

### 2.2. The Vascular Endothelium: The ‘Gatekeeper’ of Systemic Health

The vascular endothelium is a single layer of cells that covers the inner surface of the entire circulatory system, making it the largest organ in the human body. It is not just a passive physical barrier; it is a highly dynamic organ with body-wide regulatory functions and plays a key role at every stage of a blood vessel’s life. As the interface between blood and tissues, endothelial cells have extensive signalling and synthetic functions, which are crucial for organ function and overall health [26]. Their functional diversity is reflected in the precise regulation of vascular tone, coagulation, inflammation, angiogenesis and barrier permeability.

Under physiological conditions, the endothelium maintains vascular homeostasis by releasing vasodilatory factors such as nitric oxide (NO) and prostacyclin, whilst balancing vasoconstrictive factors such as endothelin-1. However, when endothelial function is impaired, this balance is disrupted, characterised by reduced NO bioavailability, increased reactive oxygen species (ROS) and the emergence of a pro-inflammatory phenotype. This is considered the initial stage in the development of cardiovascular diseases such as atherosclerosis, and represents a pivotal link between various metabolic disorders and systemic vascular diseases [27,28].

**Figure 1:**
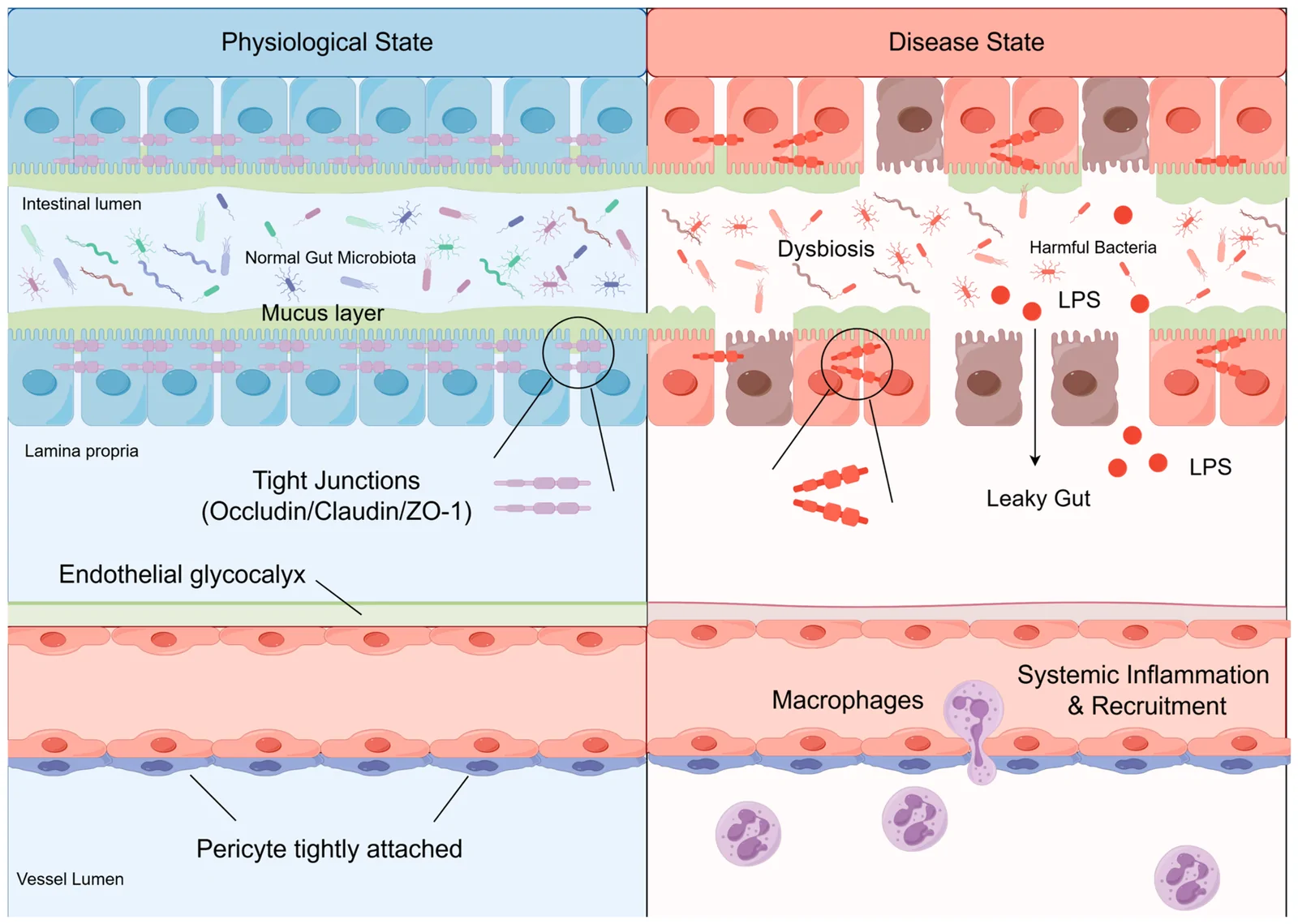
Disruption of the intestinal epithelial barrier and gut–vascular barrier (GVB) in pathological states. Left panel (Healthy): Under homeostatic conditions, the symbiotic microbiota resides within a protective mucus layer. Intestinal epithelial cells maintain integrity via tight junction proteins (occludin, claudin, ZO-1). In the submucosal microvessels, an intact gut–vascular barrier (GVB) characterised by a continuous endothelial glycocalyx and pericyte coverage prevents systemic translocation of luminal contents. Right panel (Leaky gut): Dysbiosis and mucus degradation compromise epithelial tight junctions, increasing paracellular permeability. Bacterial products (e.g., LPS) breach the epithelium and further disrupt the GVB, evidenced by glycocalyx shedding and pericyte detachment. This facilitates the entry of gut-derived pathogens into the circulation, triggering systemic immune cell recruitment (monocyte extravasation) and initiating vascular inflammation. Figure 1 was created by Figdraw. Copyright Code: YRROP0b0b0.

Especially when it comes to immune responses, the endothelium controls how immune cells move from the blood into tissues, carefully managing their entry by regulating adhesion molecules and cell junctions [29]. In systemic inflammation, such as sepsis, the activation of endothelial cells triggers a robust innate immune response to clear pathogens; however, excessive or dysregulated activation may lead to multi-organ dysfunction (Figure 1) [30].

Endothelial cells can also communicate with adipocytes, immune cells and other cells through mechanisms such as the secretion of extracellular vesicles, thereby influencing systemic energy metabolism and inflammatory states. For example, in obesity, impaired endothelium-dependent regulation of adipose tissue blood flow, alterations in endothelial cell vesicle secretion, and disrupted crosstalk with adipocytes collectively contribute to the development of metabolic disorders such as insulin resistance [31]. Consequently, the endothelium is not merely a passive conduit for substances in the circulation, but an active participant in the regulation of local and systemic metabolic homeostasis [32].

Due to its unique anatomical location, the vascular endothelium is the primary target for ‘danger signals’ of intestinal origin (such as lipopolysaccharides and bacterial metabolites) once they enter the circulation [33]; this loss of endothelial barrier function is the central link connecting gut dysfunction with systemic vascular and organ damage.

The integrity of the endothelial barrier depends on the precise regulation of intercellular junctions, cytoskeletal dynamics and cell–matrix adhesion [34]. When barrier function is disrupted, vascular permeability increases abnormally, which can lead to tissue oedema and abnormal infiltration of immune cells, and promote the development of various diseases such as atherosclerosis and cancer metastasis [35]. Thus, maintaining endothelial barrier function is crucial for preventing the spread of gut-originating inflammation throughout the body.

In summary, as a highly heterogeneous and active regulatory system, the health of the vascular endothelium serves as the cornerstone of systemic cardiovascular health, metabolic balance and immune homeostasis; endothelial dysfunction arising from any cause may act as a trigger and amplifying factor for systemic diseases [26,36].

### 2.3. The Gut Microbiota and Its Metabolites Are Key Signalling Molecules

Short-chain fatty acids (SCFAs) produced by the gut microbiota through fermentation of dietary fibre—particularly butyrate, propionate and acetate—have been reported in preclinical studies to act as beneficial signalling molecules in the gut–vascular dialogue. They provide energy to colonic epithelial cells and have been shown in vitro and in animal models to enhance intestinal barrier integrity; they are also absorbed into the portal circulation and can act on vascular endothelial cells [37]. However, the extent to which these effects translate into clinically meaningful vascular protection in humans remains incompletely defined.

Mechanistic studies, largely in cell and animal models, suggest that butyrate may improve endothelial function by inhibiting histone deacetylase activity, promoting regulatory T-cell differentiation, and alleviating oxidative stress [38]. In atherosclerosis models, butyrate has been reported to exert anti-fibrotic and anti-inflammatory effects via AhR/Nrf2 signalling [39]. These findings support a plausible protective role, but human causal evidence is still limited. Furthermore, SCFAs can stimulate endothelial cells to form tubular structures in vitro, promote placental vascularisation, and prevent placental growth restriction and vascular dysfunction caused by maternal gut microbiota depletion or malnutrition in in vivo models [40]. In acute respiratory distress syndrome (ARDS) models, supplementation with butyrate-loaded nanoparticles restores gut microbiota balance and improves pulmonary endothelial barrier function by inhibiting protein tyrosine phosphatase non-receptor type 1 (PTPN1)-mediated inflammation [41]. This evidence suggests that SCFAs, as key metabolites produced by the gut microbiota, form a vital chemical bridge linking gut health and vascular homeostasis by acting directly on endothelial cells.

However, another class of metabolites produced by the gut microbiota, such as trimethylamine N-oxide (TMAO), acts as a ‘harmful signal’ that promotes vascular endothelial dysfunction. TMAO is produced when dietary precursors such as choline and L-carnitine are metabolised by the gut microbiota to form trimethylamine (TMA), which is subsequently oxidised by flavin monooxygenase 3 (FMO3) in the liver [42]. Numerous studies have confirmed that elevated circulating TMAO levels are independent risk factors for endothelial dysfunction, platelet hyperreactivity and atherosclerosis [43], with the mechanism involving the induction of oxidative stress, inflammatory responses and cell death in endothelial cells. However, whether TMAO is a causal mediator in humans or primarily a marker of dysbiosis and impaired renal/metabolic function requires further validation. In bovine aortic endothelial cells, TMAO impairs the purinergic-stimulated increase in intracellular calcium and nitric oxide (NO) release, thereby disrupting endothelium-dependent vasodilation mechanisms [44]; in models of ageing and hypertension, TMAO promotes age-related endothelial dysfunction by increasing vascular superoxide production, reducing the activity of endothelial nitric oxide synthase (eNOS) and the bioavailability of NO [45]; furthermore, TMAO also stimulates hepatocytes to release exosomes rich in specific microRNAs (miRNAs); upon uptake by vascular endothelial cells, these exosomes further exacerbate endothelial inflammation and dysfunction by activating the nuclear factor κB (NF-κB) signalling pathway [46]. In the context of diabetic retinopathy, TMAO acts synergistically with hyperglycaemia to exacerbate the proliferation, migration and tubule formation capabilities of human retinal microvascular endothelial cells, whilst simultaneously disrupting the expression of tight junction proteins and increasing the permeability of the blood-retinal barrier [47]. Consequently, as a key pathological mediator in the gut–vascular axis, TMAO levels can serve as a biomarker for cardiovascular disease risk and represent an important target for intervention [48].

In addition to SCFAs and TMAO, the gut microbiota produces a range of other bioactive metabolites, such as secondary bile acids and tryptophan derivatives, which finely regulate metabolic and inflammatory states by activating specific receptors on host cells, thereby further enriching the ‘chemical language’ of the gut–vascular dialogue [49].

Secondary bile acids are derived from primary bile acids through modification by gut bacteria; they act as ligands to activate the farnesoid X receptor (FXR) and the G protein-coupled bile acid receptor 5 (TGR5), thereby playing a central role in regulating lipid metabolism, glucose homeostasis and inflammatory responses [50,51]. In atherosclerosis, gut microbiota-mediated changes in the composition of secondary bile acids have a critical impact on disease progression [38]; on the other hand, the metabolism of tryptophan by the gut microbiota produces various indole derivatives, such as indole-3-propionic acid (IPA) and indole-3-acetaldehyde. These metabolites exert their effects primarily by activating the aryl hydrocarbon receptor (AhR) [52].

However, its effects are complex and even contradictory. For example, studies have found that IPA impairs purine-stimulated NO release from endothelial cells, which may have a negative impact on vascular tone [53]; conversely, in a model of hepatic sinusoidal obstruction syndrome, a reduction in microbial tryptophan metabolites is associated with the disease, whilst supplementation with these metabolites can alleviate oxidative damage to hepatic sinusoidal endothelial cells by activating the AhR/Nrf2 pathway [39]. Furthermore, other microbial metabolites, such as α-hydroxyisobutyric acid (HIBA), have been found to promote atherosclerosis by inducing reactive oxygen species production and triggering inflammation in vascular endothelial cells via the activation of the thioxanthin-interacting protein (TXNIP)/NOD-like receptor heat shock protein domain-related protein 3 (NLRP3) inflammasome pathway [54,55].

**Figure 2:**
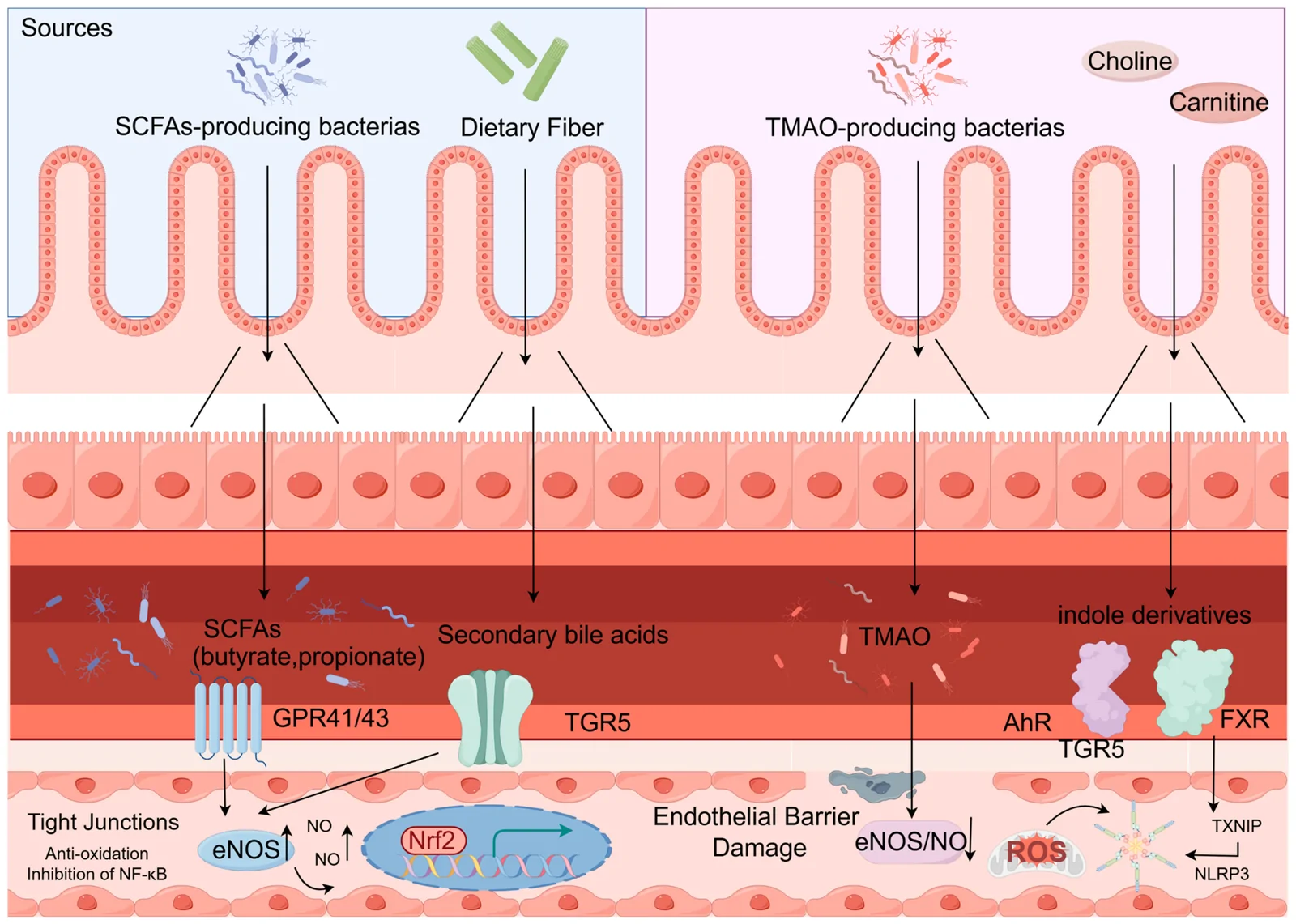
Bidirectional regulatory network of gut microbiota-derived metabolites on vascular endothelial function via specific host receptors. The figure illustrates a top-down signalling cascade from microbial metabolites to endothelial outcomes. Protective pathway (Left/Green): Beneficial microbiota ferment dietary fibres to produce short-chain fatty acids (SCFAs, e.g., butyrate/propionate), which activate GPR41/43. Concurrently, specific secondary bile acids (e.g., UDCA) signal through TGR5. These cascades converge to enhance endothelial nitric oxide synthase (eNOS)/NO bioavailability, preserve tight junction integrity, and inhibit NF-κB-mediated inflammation. Pathogenic pathway (Right/Red): Pathobionts generate detrimental metabolites, including trimethylamine N-oxide (TMAO) and α-hydroxyisobutyric acid (HIBA); certain indole derivatives act mainly via AhR, whereas bile acids act via FXR/TGR5. Receptor–metabolite assignments are context-dependent. Secondary bile acids (e.g., DCA) also contribute to this pathway. These signals drive reactive oxygen species (ROS) bursts, activate the TXNIP/NLRP3 inflammasome, suppress eNOS activity, and upregulate adhesion molecules, ultimately culminating in endothelial barrier disruption and dysfunction. Figure 2 was created by Figdraw. Copyright Code: WAWPUb74a4.

Together, these studies reveal a complex regulatory system comprising a diverse array of microbial metabolites and their receptor signalling networks (Figure 2), which collectively shape the host’s vascular endothelial phenotype and play a central role in the pathogenesis of cardiovascular disease.

The biological effects of microbial metabolites are highly context-dependent and should not be interpreted as uniform or directly causal. SCFAs can exert divergent effects depending on concentration, receptor expression (e.g., GPR41/43), tissue type, and disease state; in some settings they are protective, whereas in others they may contribute to metabolic dysregulation [56,57,58]. TMAO associations with cardiovascular disease may be influenced by renal function, diet, inflammation, and medication use [59,60], and TMAO may be a risk marker as well as a mediator [61]. Tryptophan metabolites activate AhR in a context-dependent manner and may be either protective or harmful [62,63,64]. Secondary bile acids have highly variable biological effects depending on their specific molecular structure and receptor activation profile (e.g., FXR vs TGR5) [65,66]. Therefore, metabolite–disease associations should not be equated with direct causality without further evidence.

## 3. Pathological Mechanisms of Gut–Vascular Axis Dysregulation: A Bidirectional Self-Amplifying Network

The pathological activation of the gut–vascular axis should not be understood as a linear sequence of separate events, but as a bidirectional self-amplifying network. On the one hand, dysbiosis and impaired intestinal barrier integrity—especially disruption of the epithelial barrier and the GVB—allow LPS, bile acids, indole derivatives and other microbial products to translocate into the portal and systemic circulation, and increase the availability of TMA precursors, which are subsequently converted to TMAO in the liver. These gut-derived signals activate innate immune cells and endothelial cells, mainly through TLR4/NF-κB and related pathways, leading to oxidative stress, increased vascular permeability and endothelial dysfunction. In the reverse direction, endothelial dysfunction and vascular inflammation are not merely end points: they further compromise the GVB and microvascular barrier, promote immune cell infiltration, and sustain systemic low-grade inflammation. This reverse arm exacerbates intestinal barrier injury and creates a feed-forward loop in which gut-derived damage signals and vascular dysfunction reinforce each other.

### 3.1. ‘Leaky Gut’ and Endotoxemia

Increased intestinal permeability, commonly known as ‘leaky gut’, is a key factor in the development of endotoxemia. A variety of factors, including a high-fat diet, alcohol, non-steroidal anti-inflammatory drugs (NSAIDs) or certain disease states, can lead to downregulation or abnormal distribution of tight junction proteins in the intestine, thereby compromising the integrity of the intestinal barrier [67].

For example, in a mouse model of atherosclerosis induced by a high-fat, high-cholesterol diet, the expression of colonic tight junction proteins was significantly reduced [67]; similarly, alcohol exposure disrupts intestinal epithelial tight junctions, whilst chronic stress or the stress hormone corticosterone exacerbates alcohol-induced disruption of tight junctions and mucosal barrier dysfunction [68,69]. This impairment of barrier function allows LPS, which is normally confined to the intestinal lumen, to translocate into the portal vein and systemic circulation [70]. Once in the circulation, LPS binds to lipopolysaccharide-binding protein (LBP), thereby activating Toll-like receptor 4 (TLR4), which is primarily expressed on immune cells and endothelial cells. This triggers a robust pro-inflammatory response, such as the activation of the nuclear factor κB (NF-κB) pathway, and the release of cytokines including tumour necrosis factor-α (TNF-α) and interleukin-6 (IL-6) [71]. Studies have shown that NF-κB p65-mediated pyroptosis in intestinal epithelial cells contributes to endotoxemia-induced intestinal injury [72]. This chronic, low-level endotoxemia is the core mechanism driving systemic inflammation and persistent endothelial activation.

Chronic, low-grade endotoxemia is closely associated with the pathogenesis of various metabolic and cardiovascular diseases. Extensive evidence suggests that endotoxemia is a key driver of insulin resistance and the development of early atherosclerotic lesions. Clinical studies have shown that elevated levels of the intestinal permeability markers integrin and LBP can predict the risk of cardiovascular mortality [73]. In patients with atherosclerosis, reduced expression of the colonic epithelial Krüppel-like factor 4 (KLF4) exacerbates intestinal permeability and endotoxemia via the NF-κB/microRNA-34a axis, thereby promoting the formation of atherosclerotic plaques [67]; furthermore, in patients with isolated coronary artery dilatation, serum levels of leptin and LPS are significantly elevated, suggesting that increased intestinal permeability and low-grade endotoxemia may be the triggering mechanisms underlying its pathogenesis [74].

Endotoxemia is not confined to the cardiovascular field; intestinal barrier dysfunction and LPS translocation have also been identified as key pathological mechanisms in non-alcoholic fatty liver disease, alcoholic liver disease, acute liver failure and obesity-related metabolic disorders [75,76,77]. Furthermore, dysbiosis of the gut microbiota is also a major cause of ‘leaky gut’ and endotoxemia, and diet is the primary environmental factor influencing the gut microbiota. For example, the Mediterranean diet is believed to modulate the gut microbiota and maintain intestinal barrier function, thereby alleviating endotoxemia [70]; conversely, high-fat and high-sugar diets disrupt the balance of the gut microbiota, increase intestinal permeability, and lead to metabolic endotoxemia [78].

Once LPS enters the circulation, it activates TLR4 on immune cells and endothelial cells, triggering NF-κB activation and release of TNF-α and IL-6 [79]. These inflammatory mediators can further impair tight junction integrity and GVB function, thereby increasing intestinal and vascular permeability and allowing more LPS and bacterial products to enter the circulation [3,80,81]. At the same time, since pro-inflammatory cytokines such as TNF-α can directly increase microvascular endothelial permeability and induce increased permeability at endothelial cell junctions, they may similarly impair the function of the GVB—a barrier composed of endothelial cells, pericytes, and glial cells that regulates the entry of macromolecules into the bloodstream [82,83]. Thus, LPS-mediated endotoxemia is not a one-way consequence of intestinal barrier disruption; it is a node in a bidirectional loop: leaky gut drives endotoxemia, and endotoxemia plus endothelial dysfunction further drives leaky gut and GVB disruption, forming a self-reinforcing pathological circuit [84].

### 3.2. Dysbiosis and Metabolic Endotoxin-Induced Inflammation

Structural imbalances in the gut microbiota, known as dysbiosis, are a key driver of metabolic endotoxin-induced inflammation. This dysbiosis typically manifests as a reduction in beneficial bacteria and an increase in potentially harmful bacteria, which directly impairs intestinal barrier function and increases the production of pro-inflammatory metabolites [85].

For example, in models of obesity and insulin resistance induced by a high-fat diet, a decline in the abundance of SCFA-producing beneficial bacteria has been observed, whilst potential pathogenic bacteria, such as Gram-negative bacteria, have increased [86]. This structural alteration in itself leads to downregulation of tight junction protein expression, compromising the integrity of the intestinal epithelium [87], whilst the weakened barrier function allows bacterial endotoxins, primarily LPS, to translocate into the portal vein and systemic circulation, triggering metabolic endotoxemia [88,89]. This chronic, low-grade endotoxemia is a key driver of systemic low-grade inflammation. By activating pattern recognition receptors such as TLR4, it triggers downstream inflammatory signalling pathways including NF-κB, promoting the release of pro-inflammatory cytokines such as TNF-α and IL-6, thereby exacerbating endothelial damage and insulin resistance [88,90].

From a metabolic perspective, a dysbiotic gut microbiota not only produces fewer protective metabolites but also generates more harmful substances, thereby directly or indirectly exacerbating vascular damage. A notable feature of this is the reduction in protective short-chain fatty acids (SCFAs). SCFAs are produced by the fermentation of dietary fibre by gut bacteria and exert multiple effects, including anti-inflammatory action, maintenance of intestinal barrier integrity, and improvement of insulin sensitivity [91,92]. Microbiota dysbiosis leads to a decline in SCFA production, thereby weakening their protective effects on endothelial cells [93]; simultaneously, the production of harmful metabolites such as TMAO increases. TMAO’s precursor, TMA, is primarily produced by gut microbes metabolising dietary substances such as choline and carnitine, and is subsequently oxidised to TMAO in the liver [91]. Elevated TMAO levels are closely associated with an increased risk of atherosclerosis and thrombosis; the mechanisms involved include the promotion of foam cell formation, enhanced platelet activation, and impairment of cholesterol reverse transport [91,94]. Furthermore, dysbiosis can lead to alterations in the secondary bile acid profile; for example, an increase in the proportion of pro-inflammatory bile acids such as deoxycholic acid, and a decrease in anti-inflammatory bile acids such as ursodeoxycholic acid, further disrupting the host’s metabolic and immune homeostasis [93,95].

Dysbiosis also profoundly affects bile acid metabolism and indirectly impairs vascular health by altering the signalling pathways of bile acid receptors such as the FXR. FXR is a key nuclear receptor that regulates bile acid synthesis, glucose and lipid metabolism [96]; in a state of dysbiosis, an abnormal bile acid profile can affect the activation of FXR in the gut. Studies have shown that the specific depletion of FXR ligands or the receptor itself in intestinal epithelial cells exacerbates diet-induced metabolic dysregulation [97]. Dysbiotic gut microbiota may interfere with FXR signalling by altering bile acid composition, leading to systemic glucose metabolism abnormalities and insulin resistance, whilst simultaneously promoting hepatic steatosis and inflammation [98]; these metabolic dysregulations are all significant risk factors for vascular pathology. Consequently, the microbiota dysbiosis–bile acid metabolism–FXR signalling axis constitutes a crucial bridge linking the gut ecosystem with systemic metabolism and vascular health; targeting this pathway may offer new insights into the intervention for metabolic endotoxin-induced inflammation and related vascular diseases [99].

Within this bidirectional network, microbial metabolites function as both protective and harmful signals rather than as isolated molecules [64,100,101]. SCFAs—especially butyrate, propionate and acetate—can improve endothelial function through mechanisms such as enhancing the expression of tight junction proteins and the integrity of the intestinal barrier, inhibiting the activity of histone deacetylases, and promoting the differentiation of regulatory T cells [100,102,103]. In contrast, TMAO inhibits purinergic receptor-mediated increases in intracellular calcium ion concentrations and nitric oxide (NO) release; increases the production of superoxide within blood vessels; reduces eNOS activity and the bioavailability of NO; stimulates exosomes derived from hepatocytes to activate NF-κB in endothelial cells; and disrupts tight junctions in the vascular endothelium, thereby increasing vascular permeability [42,44,101,104]. Secondary bile acids can activate FXR and TGR5 and regulate lipid metabolism, glucose homeostasis, and inflammatory responses; gut microbiota dysbiosis alters the bile acid profile, thereby disrupting FXR signalling, glucose metabolism, insulin sensitivity, and hepatic steatosis, and disrupting the composition of secondary bile acids, which in turn affects metabolic diseases [105,106,107].

Indole derivatives derived from tryptophan primarily exert their effects through the AhR and exhibit context-dependent effects: IAAld and IAA activate the AhR/Nrf2 pathway and alleviate oxidative stress-induced damage in hepatic sinusoidal endothelial cells; IPA, IAA, and IAld improve colitis by acting through the AhR/Nrf2 pathway and inhibiting NF-κB. Additionally, indole-3-acetic acid protects pulmonary microvascular endothelial barrier function by activating USP40 and reducing the ubiquitination of HSP90β [39,108,109,110]. However, other indole derivatives may exert harmful effects depending on the biological context, and the net effect of tryptophan metabolism on the vasculature cannot be generalised across all indole derivatives or disease states.

Therefore, dysbiosis may tend to shift the balance from protective short-chain fatty acids (SCFAs) and anti-inflammatory bile acids toward harmful TMAO, pro-inflammatory bile acids, and harmful indole derivatives; this imbalance, in turn, triggers immune activation, oxidative stress, and endothelial dysfunction.

### 3.3. Cross-Talk Within the Immune System and Chronic Inflammation

As the body’s largest immune organ, the gut microbiota plays a central role in shaping the host immune system [111].

An imbalance in intestinal immune homeostasis, such as a disruption in the ratio of Th17 cells to regulatory T cells (Tregs), is a key factor in the development of chronic inflammation [112]; this local immune dysregulation is not confined to the intestine but can also trigger systemic activation of immune cells. For example, in inflammatory bowel disease (IBD), chronic intestinal inflammation can impair epithelial and vascular barrier function, allowing gut-derived antigens and bacterial products such as LPS to translocate into the systemic circulation, thereby triggering systemic inflammation [113,114]. These circulating pro-inflammatory signals, particularly via pathways such as TLR4, can activate and maintain innate immune cells—such as monocytes and macrophages—in a persistent pro-inflammatory state [115]. Activated immune cells subsequently infiltrate the vascular wall, directly attacking endothelial cells and exacerbating local vascular inflammatory responses through the secretion of large quantities of pro-inflammatory cytokines [116,117]. This endothelial dysfunction and vascular inflammation constitute the initiating and central mechanisms of cardiovascular diseases such as atherosclerosis [118].

These activated immune cells, particularly macrophages derived from monocytes, play multiple roles in the process of infiltration and activation of the vascular wall—not only do they directly secrete pro-inflammatory factors that damage the endothelium, but they also take up large quantities of oxidised low-density lipoprotein, transforming into foam cells, which is a hallmark event in the formation of atherosclerotic plaques [119].

Research has further revealed that dysbiosis of the gut microbiota and alterations in its metabolites—such as a reduction in SCFAs and an increase in TMAO—can influence the inflammatory phenotype and function of immune cells by modulating their metabolism and epigenetic reprogramming, thereby persistently amplifying the inflammatory response in distal vascular sites and promoting plaque development [120,121]. For example, tryptophan derivatives produced by gut microbiota metabolism can disrupt the homeostasis of cerebral microvasculature by activating the aromatic hydrocarbon receptor (AhR) signalling pathway, thereby promoting oxidative stress and inflammation; this also applies to the systemic vascular system [64]. Consequently, gut immune dysbiosis establishes a direct link with vascular pathology by influencing the phenotype and function of circulating immune cells.

Further evidence suggests that there may be direct migration of immune cells between the gut and the vasculature, forming an immunological connection between the gut and the vasculature. Gut-derived immune cells or their precursors may migrate via the circulatory system to specific sites such as the vascular intima [122]. For example, in Crohn’s disease, abnormalities in the mesenteric compartment are closely associated with intestinal inflammation and disease progression, suggesting that the mesentery acts as a hub that may mediate signal transduction and cell migration of immune cells to the whole body, including the vascular system [123]. Furthermore, intestinal endothelial cells themselves are key participants in immune regulation. Intestinal microvascular endothelial cells engage in cross-talk with immune cells such as innate lymphoid cells of group 3 (ILC3) via molecules such as endothelin-1 (ET-1), regulating the latter’s metabolic adaptation and the production of interleukin-22 (IL-22), thereby influencing the state of intestinal and systemic immune inflammation [124].

When the gut–vascular barrier (GVB) is compromised, gut microbiota and their metabolites are more readily able to enter the portal venous circulation, directly affecting the liver and, via the systemic circulation, reaching the vascular beds of distant organs; for example, in the pathogenesis of pulmonary arterial hypertension (PAH) and hepatic sinusoidal obstruction syndrome (HSOS), it has been demonstrated that LPS translocation resulting from gut dysbiosis and GVB disruption is a key mechanism exacerbating vascular inflammation and remodelling [113,125]. This inflammatory response, which originates in the gut, is mediated by immune cells and soluble mediators, and is continuously amplified within the vascular wall, constitutes the core pathological basis of the immune crosstalk within the gut–vascular axis.

Thus, immune activation, oxidative stress and endothelial dysfunction are not separate outcomes but mutually reinforcing components of the same feed-forward gut–vascular axis loop.

### 3.4. Bidirectional Interaction Between Gut Dysfunction and Endothelial Disease

The core of the gut–vascular axis is the bidirectional interaction between gut dysfunction and endothelial disease. In the gut-to-vessel direction, dysbiosis can lead to impaired intestinal barrier function, allowing the endotoxin LPS to enter the bloodstream and trigger an inflammatory response. At the same time, the gut microbiota produces trimethylamine (TMA) through the metabolism of choline, lecithin and L-carnitine; TMA is subsequently converted into trimethylamine oxide (TMAO) in the liver by flavin monooxygenase 3 (FMO3). Furthermore, the gut microbiota is involved in the metabolic conversion of bile acids, generating a variety of secondary bile acids [126]. LPS activates TLR4/NF-κB on endothelial cells and immune cells, increasing TNF-α, IL-6, ROS, and adhesion molecules (VCAM-1, ICAM-1), reducing eNOS activity and NO bioavailability, and promoting monocyte adhesion and endothelial permeability [127,128]. TMAO directly impairs endothelial NO release, increases superoxide, and disrupts tight junction proteins [129,130,131]. Dysbiosis also reduces protective SCFAs, further weakening endothelial defence [126]. In the vessel-to-gut direction, endothelial dysfunction and vascular inflammation increase microvascular permeability, compromise the GVB, and promote immune cell infiltration into the intestinal wall [116]. This reverse arm exacerbates intestinal barrier injury, allows more microbial products to enter the circulation, and sustains systemic low-grade inflammation. Thus, gut dysfunction and endothelial disease form a self-reinforcing loop: leaky gut drives endotoxemia and endothelial injury, and endothelial dysfunction further impairs the GVB and intestinal barrier, perpetuating the cycle. This bidirectional interaction is the central therapeutic target of combined gut- and endothelium-directed interventions.

## 4. Intervention Strategies and Evidence Relating to the Gut and Vascular Endothelium

### 4.1. Drugs and Biologics Targeting the Intestinal Barrier

The restoration of intestinal barrier function is a key factor in maintaining intestinal homeostasis and systemic health. Clinically, certain essential nutrients such as glutamine and zinc preparations have been used to support the repair of the intestinal barrier [132]. These substances play a protective role in pathological conditions by providing energy substrates for intestinal epithelial cells or acting as cofactors for enzymes, thereby helping to maintain normal epithelial cell metabolism and the stability of tight junction proteins [133].

However, in the face of complex intestinal barrier damage, particularly the phenomenon of ‘leaky gut’—that is, an abnormal increase in intestinal permeability—more targeted intervention strategies are required. Current research is focusing on the development of novel drugs that act directly on key regulatory nodes of the intestinal barrier. For example, TLR4 inhibitors aim to block excessive inflammatory signals triggered by pathogen-associated molecular patterns, thereby reducing damage to tight junctions [134]; on the other hand, tight junction-modulating peptides, such as larazolide, represent another class of drugs that directly intervene in ‘leaky gut’. Studies have shown that larazotide acetate can stabilise the conformation of tight junction proteins by inhibiting the activity of myosin light chain kinase (MLCK), thereby effectively blocking the paracellular transport of macromolecules and restoring the integrity of the intestinal barrier [135]. This intervention targeting specific molecular pathways offers new hope for the treatment of diseases associated with increased intestinal permeability, such as irritable bowel syndrome and inflammatory bowel disease.

Furthermore, the optimisation of nanoscale drug delivery systems has opened up new opportunities for targeted repair of the intestinal barrier. By precisely controlling the size and distribution of nanoparticles, it is possible to enhance their interaction with the inflamed intestinal epithelium, thereby achieving drug accumulation at the target site [136,137]. These emerging targeted strategies aim to directly reinforce the intestinal barrier at the molecular level, providing more precise and effective tools for sealing ‘leaky gut’.

Biological agents, represented by TNF-α antagonists, have demonstrated exceptional efficacy in the treatment of IBD; their core mechanism of action lies in the potent suppression of abnormal local immune inflammatory responses in the intestine [138]. Clinical studies have confirmed that anti-TNF-α therapy not only effectively induces clinical remission but is also a key means of achieving endoscopic mucosal healing [139]. Mucosal healing signifies the restoration of the structure and function of the intestinal epithelial barrier, which is considered a key therapeutic objective for preventing disease recurrence and improving long-term prognosis [140].

It is worth noting that the benefits of anti-TNF-α drugs are not limited to the gastrointestinal tract. A growing body of evidence suggests that, whilst controlling intestinal inflammation, these biologics may also be associated with improvement in systemic vascular inflammation and endothelial function in some studies [141]. This phenomenon profoundly reveals the intrinsic link of the ‘gut–vascular axis’: as the body’s largest immune organ and barrier, dysfunction of the gut leads to the entry of large quantities of inflammatory mediators and bacterial products into the circulatory system, thereby activating vascular endothelial cells and triggering and sustaining systemic low-grade inflammation and endothelial dysfunction. This constitutes the common pathological basis for various vascular lesions, including atherosclerosis and cardiovascular disease [142]. Consequently, effectively controlling inflammation at the ‘source’—IBD—through anti-TNF-α drugs can reduce the spillover of pro-inflammatory cytokines into the systemic circulation, thereby indirectly alleviating attacks on and damage to the vascular endothelium [143].

The value of this strategy of intervening at the source lies in its potential to simultaneously address intestinal diseases and their associated systemic complications [141], further supporting the notion that targeted treatment of intestinal inflammation holds significant theoretical and practical implications for improving systemic vascular health. In the future, as our understanding of the gut–vascular axis deepens, the development of novel combination therapies that both promote mucosal healing and specifically improve endothelial function will become a highly promising area of research.

Thus, gut-directed interventions range from established IBD therapies to early clinical and preclinical strategies; their ability to improve hard cardiovascular outcomes through the gut–vascular axis remains unproven.

### 4.2. Therapies Targeting Vascular Endothelial Function

These endothelial-targeted therapies are clinically established for their respective cardiovascular or metabolic indications, but their GVA-specific mechanisms are largely inferred or preclinical and should not be presented as proven gut–vascular axis interventions.

#### 4.2.1. Statins

Endothelial dysfunction is a common pathological basis for various cardiovascular diseases; one of its characteristics is reduced eNOS activity and the consequent decrease in NO bioavailability [144]. As a cornerstone of endothelial-targeted therapy, the value of statins extends far beyond lipid-lowering effects. Evidence suggests that statins exert a profound influence on vascular endothelial function through their multifaceted mechanisms, including anti-inflammatory and antioxidant effects, as well as the direct promotion of endothelial nitric oxide synthase (eNOS) activity [145]. Furthermore, statins such as atorvastatin have demonstrated the ability to inhibit endothelial cell proliferation and angiogenesis in lung-cancer-associated pleural-effusion-induced angiogenesis by modulating FXR, further illustrating their vasoprotective effects beyond lipid-lowering [146]; In atherosclerosis models, the monoamine oxidase B (MAOB) inhibitor selegiline exerts anti-atherosclerotic effects by suppressing endothelial oxidative stress and dysfunction; its mechanism partly involves the regulation of the gut microbiota [147]. However, selegiline is not a statin, and its GVA-specific effects remain preclinical. It is discussed here as an example of a non-statin pharmacological intervention that may act at the interface of endothelial function and gut microbiota, rather than as an established gut–vascular axis therapy.

As dysbiosis of the gut microbiota is closely associated with endothelial dysfunction, and microbial metabolites such as TMAO and LPS can impair endothelial function [148], statins may protect the endothelium by indirectly alleviating the systemic inflammation and oxidative stress induced by dysbiosis through their anti-inflammatory and antioxidant properties [149]. Consequently, statins directly improve endothelial function via the eNOS pathway and exert systemic anti-inflammatory and antioxidant effects. Their GVA-specific mechanisms are largely inferred, but they remain the cornerstone of endothelial-targeted therapy.

#### 4.2.2. Renin–Angiotensin System Inhibitors

Inhibitors of the renin–angiotensin system (RAS), including angiotensin-converting enzyme inhibitors (ACEIs) and angiotensin II receptor blockers (ARBs), are an important class of drugs for improving endothelial function.

The core mechanism lies in blocking the deleterious effects of angiotensin II (Ang II). Upon activation via its receptors (such as AT1R), Ang II induces vasoconstriction, promotes oxidative stress, triggers inflammatory responses and causes endothelial cell apoptosis, thereby impairing endothelium-dependent vasodilation. In Ang II-induced hypertensive mouse models, marked vascular dysfunction and pathological remodelling can be observed [150]; ACEIs/ARBs, by inhibiting Ang II production or blocking its receptors, can effectively reverse these harmful effects [151]. For example, in a model of LPS-induced inflammation and dysfunction in pulmonary microvascular endothelial cells, pharmacological intervention alleviated damage by inhibiting pathways such as HMGB1/RAGE or NF-κB; as these pathways are also regulated by Ang II, this suggests that ACEIs/ARBs may protect the endothelium via similar mechanisms [152,153].

Research suggests that the gut microbiota and its metabolites may interact with the renin–angiotensin system (RAS), thereby potentially influencing blood pressure regulation and endothelial function. For example, gut dysbiosis is associated with the translocation of endotoxins, such as LPS, which may lead to systemic low-grade inflammation, which in turn may exacerbate the activation of the RAS and endothelial damage [154]; furthermore, some studies suggest that certain probiotics may help alleviate pre-eclampsia, most likely by improving gut dysbiosis and endothelial dysfunction [155]. With regard to ACEI/ARB drugs, evidence as to whether they improve endothelial function by modulating the gut–vascular axis remains preliminary and is derived primarily from indirect or preclinical observations. Nevertheless, given the recognised anti-inflammatory and antioxidant properties of these drugs, it is possible—although not yet conclusively proven—that they may, at least to some extent, alleviate endothelial dysfunction associated with gut-derived inflammatory factors. Further clinical studies are required to confirm this hypothesis.

Consequently, ACEIs and ARBs are key medications for improving endothelium-dependent vasodilation and maintaining vascular health through established angiotensin II-dependent mechanisms. Their GVA-specific effects require further clinical validation.

#### 4.2.3. Antidiabetic Drugs

In recent years, antidiabetic drugs such as metformin and sodium-glucose cotransporter 2 (SGLT2) inhibitors have been shown to offer significant cardiovascular protective benefits; this is partly attributed to their endothelial protective effects, which are independent of their hypoglycaemic action, and the reduction in chronic low-grade inflammation is likely to be one of the key mechanisms underlying this effect [156,157].

Endothelial dysfunction is an early event and a key driver of diabetic vascular complications; it is closely associated with persistent hyperglycaemia, insulin resistance and the accompanying inflammatory state. In addition to improving insulin sensitivity, metformin activates AMP-activated protein kinase (AMPK), a pathway with anti-inflammatory and antioxidant properties that inhibits pro-inflammatory signalling pathways such as NF-κB, thereby reducing vascular endothelial inflammation [158].

Metformin and SGLT2 inhibitors have been shown to confer cardiovascular benefits, but their specific mechanisms of action with regard to GVA require further stratified research. Established direct vascular effects include: SGLT2 inhibitors improve endothelial function and reduce inflammation. In addition, there are indirect metabolic or anti-inflammatory effects: both agents can reduce chronic low-grade inflammation and improve metabolic parameters [158,159,160], whilst dapagliflozin modulates the gut microbiota and improves endothelial function in mice with heart failure [161].

**Figure 3:**
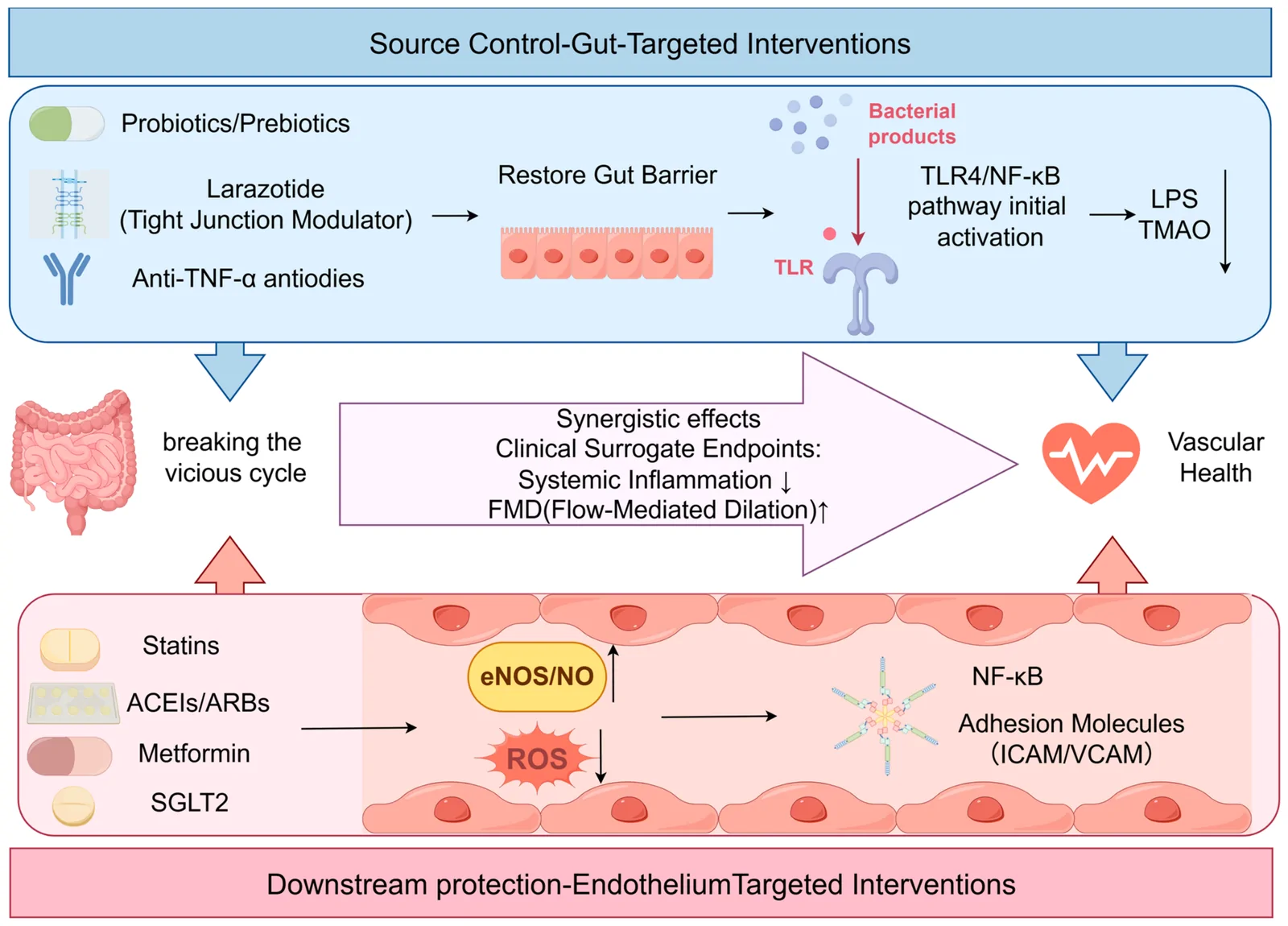
A dual-targeted “closed-loop” intervention strategy along the gut–vascular axis and its integration with precision medicine. The framework highlights synergistic effects between upstream source control and downstream vascular protection. Upper tier (Source control): Gut-directed interventions (probiotics/prebiotics, larazotide, anti-TNF-α antibodies) restore intestinal barrier integrity and inhibit the initial activation of the TLR4/NF-κB pathway, thereby curtailing the systemic translocation of LPS and reducing TMA generation and subsequent hepatic TMAO production. Lower tier (Downstream protection): Endothelium-targeted agents (statins, ACEIs/ARBs, metformin, SGLT2 inhibitors) directly enhance eNOS/NO activity, reduce ROS production, and suppress NF-κB-driven adhesion molecule expression in the vascular wall. Central synergy: The combination disrupts the vicious cycle of “leaky gut–systemic inflammation–endothelial injury”, achieving synergistic improvements in clinical surrogate endpoints, including reduced IL-6 levels and enhanced flow-mediated dilation (FMD). Figure 3 was created by Figdraw. Copyright Code: OPUOR99119.

Whether these drugs can specifically improve cardiovascular outcomes in humans via the gut–vascular axis remains inconclusive. They should not be regarded as proven gut–vascular axis interventions (Figure 3).

## 5. Theoretical Mechanisms of Synergy Between Combined Intestinal and Endothelium-Targeted Interventions

### 5.1. Synergy Between Source Control and Downstream Protection

#### 5.1.1. Gut-Targeted Interventions: Source Control

Gut-targeted interventions aim to reduce the leakage of pathogenic signalling molecules into the circulatory system at source by modulating the gut microbiota and restoring intestinal barrier function, thereby alleviating the burden of attack on endothelial cells.

As mentioned earlier, dysbiosis of the gut microbiota is a central pathological feature of various vascular diseases, and the harmful metabolites it produces are key mediators in the impairment of endothelial function [162]. For example, in chronic kidney disease, dysbiosis leads to the accumulation of TMAO, which not only exacerbates the risk of vascular calcification but also influences bone metabolism via the kidney–bone–vascular axis, highlighting the importance of the gut as the ‘source’ of pathogenic signals [5]; the integrity of the intestinal barrier, particularly the GVB, serves as a crucial defence against the entry of these harmful substances into the bloodstream.

Studies have shown that intestinal ischemia/reperfusion injury, infection with enterotoxigenic Escherichia coli, or a high-fat diet can all lead to disruption of the gut–vascular barrier (GVB), manifested by reduced expression of tight junction proteins and increased levels of endothelial permeability markers. This facilitates the translocation of endotoxins, such as LPS, and bacteria into the bloodstream, thereby triggering systemic low-grade inflammation and endothelial damage [163,164,165,166].

Consequently, gut-targeted interventions—such as the use of probiotics, prebiotics, postbiotics or specific drugs to target the Wnt/β-catenin signalling pathway—aim to restore the balance of the gut microbiota and enhance the integrity of the intestinal epithelial and vascular barriers, thereby reducing attacks on the endothelial system at the ‘source’ [13,164].

#### 5.1.2. Endothelial-Targeted Interventions: Downstream Protection

Endothelial-targeted interventions, on the other hand, focus on enhancing the endothelial cells’ own defensive, reparative and homeostatic capabilities, thereby improving their resistance to residual damage signals and their capacity for recovery.

Endothelial dysfunction is a common pathological basis for various vascular diseases, and endothelial intervention strategies aim to directly protect or repair endothelial cells. For example, studies have demonstrated that extracellular vesicles derived from mesenchymal stem cells can alleviate vascular endothelial barrier dysfunction following haemorrhagic shock or trauma by preserving tight junctions and focal adhesions between endothelial cells [167,168,169]. Furthermore, drugs such as beprastolide can protect intestinal endothelial barrier function in chronic kidney disease models by improving intestinal microcirculation and regulating the balance of reactive oxygen species and nitric oxide [170]; certain natural compounds, such as quercetin and kaempferol, have also been shown to directly enhance the defensive capacity of endothelial cells by inhibiting signalling pathways such as NF-κB or VEGF/Akt/p38, thereby mitigating high-glucose-induced or cytokine-induced damage to the intestinal microvascular endothelial barrier and abnormal angiogenesis [171,172]. The core of these interventions lies in acting directly on endothelial cells to strengthen their structural integrity and enhance their anti-inflammatory and antioxidant capabilities, thereby improving their ‘downstream’ resistance to residual damaging factors from the circulation.

Combining gut-targeted and endothelial-targeted interventions could, in theory, produce complementary effects between “prevention” and “treatment”, with results potentially superior to those of a single intervention; however, this should be described as a potential additive or synergistic interaction, not as established synergy. This synergistic strategy is based on a holistic pathophysiological framework of the gut–vascular axis. When the intestinal barrier is compromised, a flood of pathogenic signals enters the circulation; even if endothelial cells possess a certain capacity for repair, they may become decompensated due to an excessive attack load. Conversely, if endothelial defences are strengthened without addressing the source of leakage, the damage signals will persist, rendering endothelial repair efforts less effective than they might otherwise be. Consequently, the complementary effects arising from combined interventions [173,174] enable a more systematic combined approach across both upstream and downstream components of the gut–vascular axis. By simultaneously reducing the source of damage and enhancing repair capacity, this approach offers a promising new integrated therapeutic strategy for conditions associated with gut–vascular axis dysfunction, such as atherosclerosis, hypertension and vascular dementia [6,175].

### 5.2. Multilevel Inhibition of Inflammatory Pathways

#### 5.2.1. Intestinal Interventions Suppress the Initial Activation of Inflammatory Pathways Such as TLR4/NF-κB by Modulating the Microbiota and the Intestinal Barrier

As the primary interface between the body and the external environment, the integrity of the intestinal barrier serves as the ‘first line of defence’ against systemic inflammation. Disruption of the intestinal barrier—particularly damage to the gut–blood barrier (GVB)—can lead to the translocation of gut microbiota and their metabolites into the bloodstream, thereby triggering a systemic inflammatory cascade [176]. The core mechanism underlying this process involves the activation of TLR4 and the downstream NF-κB signalling pathway.

Similarly, in inflammatory bowel disease (IBD), chronic intestinal inflammation leads to impaired epithelial and vascular barrier function, allowing pathogen-associated molecular patterns such as LPS of intestinal origin to enter the systemic circulation, thereby driving systemic inflammation through activation of the TLR4/NF-κB pathway [177]. Gut-targeted interventions, such as probiotics, prebiotics or specific medications, can suppress this initial inflammatory trigger by restoring gut microbiota homeostasis and enhancing barrier function. Studies have shown that supplementation with specific probiotics can enhance intestinal barrier function and reduce serum LPS levels, thereby alleviating subsequent systemic inflammatory responses [178].

Consequently, by regulating the gut microbiota and repairing the intestinal barrier, it is possible to effectively inhibit the initial activation of key inflammatory pathways such as TLR4/NF-κB at the source, thereby breaking the vicious cycle of chronic inflammation.

#### 5.2.2. Endothelial-Targeting Drugs Can Further Inhibit Subsequent Stages of the Same Inflammatory Pathway at Both the Systemic and Vascular Wall Levels

When impaired intestinal barrier function allows inflammatory mediators to enter the circulation, vascular endothelial cells become the next key target in the inflammatory cascade.

Endothelial dysfunction is not only a hallmark of vascular disease, but also a key link between intestinal inflammation and systemic pathology [179]. Metabolic products of intestinal origin, such as LPS and TMAO, which enter the circulation, can act directly on vascular endothelial cells, continuously activating the TLR4/NF-κB pathway, promoting the expression of adhesion molecules, increasing vascular permeability and recruiting white blood cells, thereby amplifying the inflammatory response at the level of the vascular wall [118]. At this stage, interventions targeting the endothelium are particularly important.

As previously discussed, statins, as classic lipid-lowering drugs, have been shown to exert multiple anti-inflammatory and endothelium-protective effects. Their mechanisms of action include inhibiting the activation of NF-κB in endothelial cells, reducing the production of pro-inflammatory cytokines, enhancing the bioavailability of nitric oxide (NO), and stabilising the endothelial glycocalyx [118].

Furthermore, in an LPS-induced acute lung injury model, the microbial metabolite indole-3-acetic acid (IAA) reduced the ubiquitination levels of heat shock protein 90β (HSP90β) by activating the deubiquitinating enzyme USP40, thereby protecting the pulmonary microvascular endothelial barrier function and suppressing the inflammatory response [109].

This evidence suggests that endothelial intervention can effectively curb the subsequent activation and amplification of the same inflammatory pathway (such as TLR4/NF-κB) at a ‘second line of defence’ during the process by which inflammatory signals are transmitted from the gut to the systemic vascular system.

#### 5.2.3. The Combined Inhibition of Different Nodes in the Inflammatory Cascade May Produce a Stronger Anti-Inflammatory Effect

The essence of chronic inflammation lies in the formation of a self-reinforcing vicious cycle of inflammatory signals between different organs and tissues. The concept of the gut–vascular axis reveals the close coupling between the gut and the vascular system in the spread of inflammation [176].

Interventions targeting either the gut or the vascular system alone may be insufficient to completely break this cycle, as inflammatory signals may persist or rebound via alternative pathways even after partial inhibition at one node [180]. Therefore, combining gut-targeted and endothelial-targeted interventions to achieve multi-level blockade at different nodes of the inflammatory cascade could, in theory, produce additive or, if formally demonstrated, synergistic anti-inflammatory effects.

For example, in a hypertensive model, short-term exposure to cold hypoxia disrupts the microbiota–gut–brain axis, activating the LPS-TLR4/MyD88/NF-κB signalling pathway in the gut and brain, leading to neuroinflammation and sympathetic activation [181]. Supplementation with γ-aminobutyric acid (GABA) can lower blood pressure, restore the function of this axis and inhibit the aforementioned signalling pathway; this in itself demonstrates a comprehensive effect that simultaneously modulates multiple levels, including the gut microbiota, the nervous system and the vasculature. This combined strategy, which acts simultaneously at both upstream and downstream levels, holds promise for more thoroughly interrupting the inflammatory signalling cascade, thereby providing a new and more effective therapeutic paradigm for the treatment of diseases closely associated with chronic inflammation, such as IBD, atherosclerosis and metabolic disorders.

The mechanisms described in this section are theoretical and based mainly on preclinical or early clinical evidence; they are not established clinical effects.

## 6. Research Evidence on Combined Interventions

### 6.1. Evidence for Combined Effects in Animal Models

In a high-fat diet-induced atherosclerosis model using ApoE−/− mice, the combination of probiotics and statins demonstrated efficacy superior to statins alone in reducing plaque area and lowering systemic inflammatory markers. This finding supports a greater combined effect in animals but cannot be directly extrapolated to clinical hard endpoints. Importantly, because the study did not directly compare the combination with probiotic monotherapy or use a formal interaction test, this result cannot establish true synergy; additive or independent effects remain possible. Animal model studies provide key evidence for understanding synergistic interventions in the gut–vascular axis [182]. The findings suggest that modulating intracellular homeostatic mechanisms (such as mitochondrial autophagy) is a crucial pathway for improving vascular health, whilst gut microbiota may influence these intracellular processes through their metabolites. Furthermore, research on the relationship between gut microbiota and vascular ageing has found that faecal microbiota transplantation (FMT) from young mice to aged mice can significantly reverse vascular dysfunction and metabolic damage, improve endothelium-dependent vasodilation in arteries, and alleviate inflammation and telomere dysfunction in the aorta and gut [183].

These findings directly support strategies aimed at improving vascular function through the modulation of the gut microbiota. Mechanistically, this improvement may be associated with the restoration of intestinal barrier function and the activation of the AMPK/SIRT1 signalling pathway. It is therefore evident that, in atherosclerosis models, combining interventions aimed at improving gut microbiota composition with those designed to directly protect blood vessels may, through multi-target combined effects, more effectively reduce plaque formation and systemic inflammation than some monotherapies [184]. However, unless both monotherapies and the combination are compared in the same experiment with formal interaction testing, these findings should be described as additive or potentially synergistic rather than as proven synergy.

To validate the dual regulatory effect of combined interventions on the gut–vascular axis, some animal studies have employed a comprehensive, multi-level assessment approach.

Firstly, intestinal barrier integrity and permeability are key assessment indicators. For example, in a model of intestinal ischaemia–reperfusion (I/R) injury, changes in intestinal permeability and motility were assessed, along with improvements in these parameters following drug treatment [185]. In addition, the detection of markers of systemic inflammation and oxidative stress is crucial. Studies have assessed the systemic anti-inflammatory and antioxidant effects of interventions by measuring serum levels of LPS, inflammatory cytokines and oxidative stress markers [182]. Furthermore, direct assessment of vascular function is central. This includes evaluating endothelium-dependent relaxation (EDR) via in vitro vascular loop tension measurements [183] and assessing haemodynamic parameters using ultrasound or catheterisation techniques. In studies of hypertension, reductions in systolic and diastolic blood pressure following intervention are also important indicators of improved vascular function [186].

By integrating these multidimensional data spanning from the gut to the vascular system [186,187], the study suggested that a combined intervention can simultaneously improve intestinal barrier function, alleviate systemic inflammation and oxidative stress, and ultimately restore vascular homeostasis. Nevertheless, the results of other animal experiments should not be classified as direct combined evidence. For instance, an intervention targeting the gut that results in endothelial improvement does not constitute a combined intervention acting simultaneously on both the gut and endothelium. These distinctions are important, as they limit the strength of inferences regarding combination therapy.

### 6.2. Evidence from Clinical Studies

Clinical studies that directly combine gut- and endothelium-directed interventions are very limited. To improve precision, we distinguish randomised controlled trials (RCTs), observational studies, mechanistic studies, and narrative reviews; only RCTs with appropriate comparators can support clinical efficacy claims.

In metabolic syndrome, small short-term studies have suggested that probiotic supplementation may improve endothelial function. At present, no large RCT has directly compared a gut-directed intervention plus an endothelium-directed intervention with both corresponding monotherapies in metabolic syndrome. Small studies using surrogate endpoints such as flow-mediated dilation (FMD) and IL-6 remain hypothesis-generating and cannot establish synergy [188].

The MaPLE trial was a randomised controlled trial in older adults with increased intestinal permeability. A polyphenol-rich diet reduced serum and faecal calprotectin and increased ZO-1 expression [189]. However, this was a gut-targeted dietary intervention; it did not concurrently target endothelial cells, did not compare combination therapy with endothelium-directed monotherapy, and did not perform interaction testing. It therefore cannot be considered a combined gut–endothelium intervention or evidence of synergy.

In heart failure, the combination of prebiotics with standard drug therapy has been proposed [190], but the cited reference is not a clinical trial. No published randomised trial with hard endpoints is currently available. This remains an important research gap rather than clinical evidence of combination benefit. Similarly, the negative correlation between acetate and muscle sympathetic nerve activity (MSNA) [191] is an observational association, not proof that combined therapy improves cardiovascular outcomes.

However, it must be clearly recognised that there is currently a lack of large-scale, long-term randomised controlled trials in this field to verify whether combined intervention strategies can ultimately reduce hard endpoints such as major adverse cardiovascular events (MACE). Most existing studies have small sample sizes, relatively short intervention periods, heterogeneous intervention protocols, inconsistent endpoint definitions, and rely primarily on surrogate endpoints such as FMD and inflammatory markers [188,189]. These surrogate endpoints do not fully equate to clinical benefit. In addition, long-term safety data for sustained microbiome modulation are limited, and the reproducibility of findings across different populations, diets, drugs, and healthcare settings is uncertain. Animal findings cannot be directly extrapolated because rodent models do not reproduce the full natural history of human atherosclerotic cardiovascular disease, including plaque rupture, myocardial infarction, stroke, and cardiovascular death. Future research requires rigorous multi-arm or factorial clinical trials, standardised intervention and endpoint definitions, long-term safety monitoring, and sufficient follow-up to determine whether combined therapy can improve survival and quality of life in patients with atherosclerosis, heart failure, and other conditions [192,193]. The substantial inter-individual variability in gut microbiota composition further limits reproducibility and must be addressed before precise and effective interventions can be achieved [190].

The most important gap identified in this review is the scarcity of studies that simultaneously target both the gut and the endothelium in the same experimental or clinical design and directly compare combination therapy with both corresponding monotherapies. Most evidence supports either gut-directed interventions with vascular readouts or endothelium-directed therapies with inferred gut–vascular relevance. These separate lines of evidence cannot establish that combined therapy is superior to monotherapy in humans, nor can they distinguish additive effects from true synergy.

Future studies should therefore use multi-arm or factorial designs that compare gut-directed therapy alone, endothelium-directed therapy alone, combined therapy, and placebo/standard care, with formal interaction testing to distinguish additive from synergistic effects. Such studies should measure both gut-level targets (microbiota composition, intestinal permeability, LPS, TMAO, SCFAs, bile acids, inflammatory mediators) and endothelial targets (eNOS/NO, ROS, NF-κB, VCAM-1/ICAM-1, endothelial permeability, FMD, endothelium-dependent vasodilation). Hard cardiovascular endpoints such as MACE and mortality are required before combined gut- and endothelium-directed therapy can be considered clinically established.

### 6.3. Evidence Strength and Limitations of Causal Inference

Although the gut–vascular axis is biologically plausible and supported by convergent preclinical and human observational data, several mechanistic claims remain incompletely established.

First, the term “dysbiosis” lacks a standardised definition; studies use different reference communities, ecological metrics, and thresholds, which limits cross-study comparability and patient stratification [194,195]. Second, microbial metabolite effects are frequently context-, tissue-, and concentration-dependent. SCFAs and bile acids can exert divergent effects, and TMAO may be a risk marker as well as a mediator [196,197]. In addition, enteroendocrine regulation of barrier function is supported mainly by preclinical studies; many vascular benefits of microbiota-related interventions have been demonstrated in animal models or small human trials using surrogate endpoints, not in large randomised trials with hard cardiovascular endpoints [198,199,200]. Therefore, the mechanisms described in this review should be interpreted as an integrated, evidence-based framework rather than as a set of fully validated causal pathways.

In this review, a combined gut–vascular intervention is defined as an experimental or clinical design in which at least one gut-directed intervention and at least one endothelium-directed intervention are administered concurrently, with the explicit aim of modifying both gut-derived signals and endothelial function. Studies that apply only a gut-directed intervention and measure vascular outcomes, or only an endothelium-directed intervention and infer gut–vascular relevance, are not considered direct combined interventions. To claim synergy, a study must compare the combination with both corresponding monotherapies and report a formal interaction test showing an effect greater than the sum of the individual effects.

## 7. Current Challenges and Future Treatment Directions

### 7.1. Challenges in Individualised Responses, Intervention Standardisation, Long-Term Safety, and Reproducibility

There are substantial baseline differences in gut microbiota composition between individuals, leading to highly heterogeneous responses to interventions such as probiotics and prebiotics. A ‘one-size-fits-all’ approach may prove ineffective. Clinical studies have repeatedly demonstrated significant variations in the cognitive improvement effects of the same probiotic intervention across different individuals, suggesting that baseline characteristics of the microbiota composition are key determinants of the response to intervention [201,202,203]. In dietary interventions, whilst the fibre- and polyphenol-rich Mediterranean diet generally reduces the risk of cognitive decline, its specific effects are also profoundly influenced by an individual’s initial metabolic state and gut microbiota composition [204]. Consequently, ‘one-size-fits-all’ interventions that do not take individual microbiota characteristics into account often yield limited and unpredictable outcomes, highlighting the necessity of moving towards personalised precision medicine.

Although most gut-targeted interventions are generally safe, caution is still required when combining them with specific medications, as there is a theoretical risk of infection or drug interactions. Probiotics and high-fibre diets are generally considered safe; however, in immunocompromised individuals, such as patients taking immunosuppressants, the introduction of live bacterial preparations carries a theoretical risk of causing bacteraemia or infection. Although clinical reports of such events are rare, a high degree of vigilance is still required [205,206]. In diseases involving abnormal immune activation, such as spondyloarthritis, environmental factors and dysbiosis jointly drive inflammation; inappropriate modulation of the microbiota during potent immunosuppressive therapy may disrupt immune homeostasis, leading to unpredictable consequences [207]; furthermore, certain gut microbiota metabolites possess inherent toxicity [208]. Therefore, when formulating combined treatment regimens involving the gut–vascular axis, it is essential to comprehensively assess the patient’s overall health status, particularly their immune status and organ function, in order to mitigate potential risks.

### 7.2. Future Direction: Multi-Omics-Guided Stratification and Personalised Gut–Vascular Interventions

By integrating gut metagenomic, metabolomic, host genomic and clinical phenotypic data, we aim to develop predictive models in the future to identify which patients are most likely to benefit from specific combined interventions, thereby enabling treatment stratification. It should be emphasised that the approach outlined in this section represents a future research direction rather than an established clinical paradigm. Multi-omics-based stratification remains at the research stage; there is currently a lack of prospective validation, standardised testing methods, biomarkers that meet regulatory standards, and evidence of clinical utility.

As a complex system, dysfunction of the gut–vascular axis involves interactions across multiple levels, including the gut microbiota, metabolites, the host immune system and the vascular endothelium. Consequently, traditional single biomarkers or clinical indicators struggle to accurately predict an individual’s response to specific interventions. The integration of multi-omics technologies provides a powerful tool for addressing this challenge. By simultaneously analysing the gut metagenome, metabolome, host genome and detailed clinical phenotypic data, multidimensional predictive models can be constructed. For example, in the field of chronic kidney disease, the integration of multi-omics analyses to reveal associations between the gut microbiota, uremic toxins and cardiovascular risk is considered a key direction for future nephrological practice [209].

By integrating and analysing these data layers, and utilising algorithms such as machine learning, it is possible to identify patient subgroups with a high likelihood of responding to specific combined interventions. For example, in research on metabolic syndrome, the combination of gut microbiota, epigenetic regulation and metabolomics analyses has been used for early detection and risk stratification [210]. This multi-omics-based stratified medical strategy enables a shift from a ‘one-size-fits-all’ treatment model to precision interventions targeting specific pathophysiological subtypes, thereby maximising therapeutic benefits and reducing unnecessary side effects.

Studies have shown that TMAO-induced endothelial dysfunction is time-dependent and involves specific molecular features of inflammatory and remodelling pathways [211]; consequently, continuous monitoring of TMAO levels can assess the efficacy of interventions targeting microbial metabolism [212]. Furthermore, intestinal fatty acid-binding proteins serve as specific markers of intestinal epithelial cell damage, and elevated levels indicate compromised intestinal barrier function [213]. In clinical practice, regular testing of these dynamic biomarkers enables the construction of an individualised ‘gut–vascular axis functional profile’. Through dynamic monitoring of these markers, clinicians could in future more objectively assess the early efficacy of combined interventions. If biomarker levels do not improve as expected, this indicates a need to adjust the intervention strategy, such as changing probiotic strains, adjusting drug dosages, or combining with other therapeutic approaches. This ‘monitor-adjust’ closed-loop management strategy is a key component in achieving precision-targeted treatment of the gut–vascular axis and optimising long-term patient outcomes.

## 8. Conclusions

The introduction of the gut–vascular axis concept signifies a shift in our understanding of chronic diseases—particularly major conditions such as atherosclerotic cardiovascular disease (ASCVD)—from viewing individual organ systems in isolation to focusing on the complex, dynamic networks of interactions between organs.

This review narratively synthesises the existing evidence, revealing profound and bidirectional causal links between gut dysbiosis, impaired intestinal barrier function, and vascular endothelial dysfunction, as well as systemic chronic inflammation. This axis not only provides a common pathophysiological framework for a variety of seemingly unrelated chronic diseases, but, more importantly, points towards a highly promising new therapeutic paradigm: integrated intervention strategies that simultaneously target the gut and the vasculature.

Preclinical and early clinical evidence suggests that modulating the gut microbiota through probiotics, prebiotics, dietary adjustments, or specific pharmacological interventions may reduce the translocation of pathogenic bacterial products at the ‘source’, downregulate systemic low-grade inflammation, and improve endothelium-dependent vasodilation. However, direct evidence that combined gut- and endothelial-targeted interventions provide superior clinical outcomes is still limited. Such interventions may theoretically produce additive or, if formally demonstrated, synergistic effects with traditional vascular protection strategies; however, current evidence does not establish true synergy. Mechanisms range from source control and multi-level blockade of inflammatory signals to the reintegration of host microbial co-metabolic networks. This provides a theoretical basis for further clinical validation; however, it must be emphasised that there is currently no high-quality evidence to support the notion that combined interventions can improve hard endpoints, nor is there high-quality factorial evidence demonstrating true synergy. All inferences regarding clinical benefits remain at the hypothesis-generation stage.

However, we still face significant challenges in translating this promising research paradigm into widely applicable clinical practice. The primary issue is that most of the supporting evidence still derives from animal models, small-scale, short-term observational studies in humans, or clinical trials using surrogate endpoints; there is a lack of data from large-scale, long-term, randomised controlled trials that use ‘hard endpoints’ such as cardiovascular events and all-cause mortality as evaluation criteria. Secondly, there are substantial variations in gut microbiota composition between individuals, leading to potentially vastly different responses to the same intervention; this necessitates that future treatments must become more precise and personalised. Furthermore, the safety of long-term gut microbiota modulation—particularly in terms of avoiding the unknown risks associated with potential dysbiosis—requires continuous monitoring and evaluation.

In the future, research in this field should be pursued in depth along several key directions. Firstly, well-designed combination trials should be conducted using multi-arm or factorial designs that directly compare gut-directed monotherapy, endothelium-directed monotherapy, combined therapy, and placebo/standard care. Secondly, appropriate control groups that allow both corresponding monotherapies and the combination to be evaluated within the same trial are necessary. Moreover, standardised, pre-specified definitions of synergy should be formed, including formal interaction testing to distinguish additive from synergistic effects. In addition, clinically meaningful endpoints, including hard cardiovascular outcomes such as MACE, cardiovascular mortality, and quality of life, are necessary, alongside validated surrogate endpoints; and finally, longer-term assessment of cardiovascular outcomes and safety is needed, with sufficient follow-up to detect delayed benefits or harms. These priorities should guide future trial design and the clinical translation of the gut–vascular axis framework.

## Data Availability

No new data were created or analysed in this study. Data sharing is not applicable to this article.
